# Use of Iatrogenic Lipid Emulsion and Subsequent Plasmapheresis for the Treatment of Amitriptyline Overdose

**DOI:** 10.1155/2022/1090795

**Published:** 2022-10-05

**Authors:** Ryan C. Laffin, Ashley M. Cunningham, Sean A. Fitzgerald, Donald A. Wiebe, Jeffrey A. Wells, Janice Robin Linzell, William Nicholas Rose

**Affiliations:** ^1^University of Wisconsin School of Medicine and Public Health, Department of Pathology, USA; ^2^University of Wisconsin School of Medicine and Public Health, Department of Pulmonary and Critical Care Medicine, USA

## Abstract

Plasmapheresis for the treatment of hypertriglyceridemia is relatively uncommon and mostly reported either in patients experiencing hypertriglyceridemia-induced acute pancreatitis or patients with therapy-resistant familial hypercholesterolemia. Standard therapies for hypertriglyceridemia include dietary modification and lipid-lowering medication. For severe hypertriglyceridemia, the risk of pancreatitis increases significantly as triglyceride levels increase above 1000 mg/dL, and current therapies are unable to reduce triglyceride levels rapidly enough. Here, we report a case of a 48-year-old male patient who presented to the emergency department due to an amitriptyline overdose. In addition to being started on IV sodium bicarbonate therapy, an intravenous 20% fat emulsion bolus at 1.5 mL/kg was administered followed by 0.25 mL/kg/min infusion for 4 hours as a strategy to absorb lipophilic amitriptyline. Two days posttreatment, he was noted to have substantial hypertriglyceridemia (serum triglycerides: 6,475 mg/dL). His amylase was within the normal range at 37 U/L (reference range: 20-100 U/L), his lipase was low at 40 U/L (reference range: 75-390 U/L), and he was without evidence of any clinical sequelae secondary to hypertriglyceridemia (e.g., pancreatitis). Due to the severity of his hypertriglyceridemia, plasmapheresis was initiated urgently for rapid reduction in serum triglyceride levels to prevent pancreatitis and end-organ damage. He underwent a 1-plasma volume exchange with 5% albumin as the replacement fluid. This reduced his triglyceride levels to 185 mg/dL (reference range: 3-149 mg/dL). His symptoms secondary to his amitriptyline overdose were also resolved. Here, we report a 2-step process of intravenous lipid emulsion followed by plasmapheresis for amitriptyline overdose.

## 1. Introduction

Amitriptyline is a tricyclic antidepressant (TCA) used primarily in the treatment of major depressive disorder in addition to many neurological conditions including migraine prophylaxis, chronic fatigue syndrome, and neuropathic pain [1]. In vivo, this medication has a half-life of 12.9-36.1 hours, primarily is distributed throughout the body attached to protein, and is primarily hepatically metabolized [1]. When taken in excess, amitriptyline, like other TCAs, may cause clinical sequelae including hyponatremia and altered consciousness; although, other complications including cardiac arrhythmia, seizure, and respiratory depression may occur [2].

To treat an overdose of this medication, first-line therapy is sodium bicarbonate therapy [2]. If this initial treatment fails, iatrogenic lipid emulsions have been shown to reduce TCA levels in animal and human models [3–6]. Although this therapy shows promise in the treatment of TCA and other lipid soluble medication overdose, iatrogenic lipid emulsion may lead to hypertriglyceridemia; although, these effects are expected to typically taper transiently in a few hours given the short (approximately 15 minutes) half-life of this therapy [7, 8].

In cases of severe persistent hypertriglyceridemia, few therapeutic options have been studied to rapidly reduce serum triglycerides. One option in these situations may be to use centrifugal plasmapheresis to rapidly reduce serum triglycerides. Centrifugal plasmapheresis separates the patient's blood into constituents based on density, and the component of interest can be removed and replaced with another fluid [9]. We present a patient who underwent lipid emulsion therapy secondary to an amitriptyline overdose with subsequent plasmapheresis which successfully lowered his triglyceride levels.

## 2. Case Presentation

A 48-year-old man with a history of cervical radiculopathy, ADHD, migraines, and hypertension was admitted to the emergency department after experiencing a pulseless electrical activity cardiac arrest. He was found to be unresponsive, hypotensive, and bradycardic by EMS who intubated and successfully resuscitated the patient in the field. The patient was transported to the emergency department where an ECG revealed a possible right bundle branch block. While in the department, the patient experienced another PEA cardiac arrest and was again successfully resuscitated.

After successful resuscitation, cardiac catheterization was performed which revealed no significant coronary arteriosclerosis. An echocardiogram revealed reduced left ventricular systolic function but was otherwise normal. A repeat ECG demonstrated persistent bradycardia and prolonged QRS complexes. Given these ECG findings, the patient had a temporary transvenous pacemaker placed. His hypotension was treated with fluids, norepinephrine, and epinephrine. Dopamine and dobutamine were administered to maintain adequate systolic blood pressure.

Given few cardiovascular risk factors, his cardiac arrest seemed likely to be due to a secondary etiology. On review of his outpatient medications, it was revealed that 27 capsules of amitriptyline were missing from his medication bottles. Given his presentation of altered mental status, cardiac dysfunction, and hypotension, his cardiac arrest was consistent with a TCA toxicity secondary to an amitriptyline overdose. A serum TCA level was found to be elevated at 634 ng/mL. He was started on IV sodium bicarbonate 200 mEq in dextrose 5% infusion 1250 mL (equivalent to 0.16 mEq/mL). Prior to administration of sodium bicarbonate, his ABG revealed a HCO_3_ of 18.8 mmol/L, pH of 7.44, a pO_2_ of 321 mmHg, and a PCO_2_ of 28 mmHg. After completion of the sodium bicarbonate infusion, his ABG showed HCO_3_ of 23.0 mmol/L, pH of 7.49, a pO_2_ of 133 mmHg, and a PCO_2_ of 30 mmHg.

An intravenous 20% fat emulsion bolus at 1.5 mL/kg was administered followed by 0.25 mL/kg/min infusions for 4 hours in an attempt to absorb amitriptyline. Marked improvement in systolic blood pressure was observed. No baseline triglyceride levels were available before the fat emulsion bolus was administered. Two days posttreatment, his triglycerides were noted to be elevated at 6,475 mg/dL. His amylase level at that time was 37 U/L (reference range: 20-100 U/L), and his lipase level was 40 U/L (reference range: 75-390 U/L). He had not developed any clinical signs or symptoms of pancreatitis secondary to hypertriglyceridemia over his two day hospital course.

Due to the severity of his persistent hypertriglyceridemia, plasmapheresis was initiated urgently for rapid reduction in serum triglyceride levels to prevent pancreatitis and end-organ damage. He underwent a 1-volume plasma exchange via centrifugal plasmapheresis with 5% albumin as the replacement fluid. His triglyceride level after plasmapheresis improved to 185 mg/dL.

Upon completion of this therapy, the patient's mental status and bradycardia improved. Once his EKG stabilized to normal sinus rhythm with normal intervals, his temporary transvenous pacemaker was removed. On hospital day 3, his serum triglyceride levels continued to decrease to 91 mg/dL. Of note, no postplasmapheresis amitriptyline or TCA metabolite levels were performed. On hospital day 6, he was discharged.

## 3. Discussion

Here, we present a case of severe iatrogenic hypertriglyceridemia secondary to intravenous fat emulsion therapy treated with plasmapheresis. In the setting of the patient's severely elevated triglyceride levels not significantly decreasing after two days, the rationale to use plasmapheresis was that the patient's triglyceride levels were stagnant instead of transient. The clinical team anticipated that the persistent hypertriglyceridemia may have led to the development of acute pancreatitis in the patient [10]. We found that plasmapheresis markedly and rapidly reduced serum triglyceride levels without significant clinical sequelae. Our findings are limited in that this a single drug in a single case without a control, and we cannot discern for certain the extent to which using this two-step process of a lipid emulsion followed by subsequent plasmapheresis for other lipophilic drugs (e.g., other TCAs and amiodarone) may be effective.

An obvious limitation to this report was that we cannot definitively report that we decreased the drug level as a result of these interventions given that a repeat amitriptyline and metabolite level were not performed posttherapy. However, we do postulate that in circumstances in which the stakes are high that this therapy may be used as a therapy option given failure of typical first- or second-line therapies as this therapy did reduce the patient's clinical sequelae when refractory to first line sodium bicarbonate.

Further investigation regarding the use of plasmapheresis in the setting of iatrogenic hypertriglyceridemia as prevention of hypertriglyceridemia-induced acute pancreatitis should be pursued to understand the mechanisms of therapy as well as the generalizability of these findings.

Some case reports indicate a possible utility of using a “lipid sink” to absorb lipophilic drugs. For example, one case reported a comatose patient with hypotension and resistant dysrhythmia after amitriptyline overdose who was treated with intravenous lipid emulsion therapy [5]. Based on the patient's improvement, the authors concluded that early onset of intravenous lipid emulsion therapy may be life-saving in patients with resistant hemodynamic instability due to TCA-induced severe cardiotoxicity [5].

Another case report described a patient who underwent a similar 2-step process [11]. One difference was that he received 2 plasmapheresis procedures with one per day on 2 consecutive days. After these were performed, he remained hemodynamically unstable, developed ARDS, and eventually improved enough to be discharged out of the medical unit after an interval of approximately 3 weeks. We agree with these authors that the use of this 2-step process in TCA poisoning is controversial. One reply to their article was written by a group of toxicology experts who contend that it is unclear whether intravenous lipid emulsion or plasmapheresis was beneficial, neutral, or even harmful in this case [12]. Furthermore, a review article about this topic written by an international group of toxicology experts concluded that the effect of intravenous lipid emulsion therapy in various nonlocal anesthetic poisonings is heterogenous, and the quality of evidence remains low to very low [6].

However, another case report described a patient with amitriptyline toxicity who appeared to benefit clinically from plasmapheresis alone without intravenous lipid emulsion [13]. The patient's plasma amitriptyline levels before and after the first plasmapheresis session were 4.87 *μ*g/mL and 0.229 *μ*g/mL respectively [13]. This may also address one weakness of our case, as we do not have a postplasmapheresis drug level. An evidence-based review written by a working group of experts recently published a useful clinical approach and criteria for the consideration of an extracorporeal treatment in the management of a poisonous substance [14].

In summary, we report a case of a patient who appeared to benefit clinically from a 2-step process of intravenous lipid emulsion therapy ostensibly to absorb the lipophilic drug followed by plasmapheresis to remove excess lipid and presumably the drug. Plasmapheresis was initiated urgently for rapid reduction in serum triglyceride levels to prevent pancreatitis and end-organ damage. However, further trials are necessary for validation of our findings on this subject.
